# Sabbath Hygiene

**Published:** 1876-02

**Authors:** 


### SABBATH HYGIENE.

The Sixth Avenue (horse-car) Railroad is the most profitable in the city. The cars run day and night throughout the year. The company, finding that their horses and mules could not stand this incessant labor, have for some time past had a number of what they call *seventh-day* horses, which are so used as to allow the others to have one day in seven for rest. Whether they have "seventh-day men," we are not advised: we presume not; as nothing touches the heart of a monopoly but the dollar. The loss of a mule would affect them seriously; the loss of a man not at all.

But the same fact has been abundantly proven in the House of Commons, by the examination of scientific men, in the presentation of a mass of evidence, the evidence of facts which could not be gainsayed, that a rest of one day in seven is essential to the physical well-being of man and beast.

The experiment was made during the Revolutionary War, by sending several wagon-loads of provisions to the army posts, with directions that one portion of them should travel daily, and the other should rest on Sundays. The Sunday-resting teams reached their destination first, and in good condition, the others came up later, in bad trim, and much the worse for the wear.

It is not important to argue this point at length, because it has been abundantly proven, over and over again, by theory and by practice, that periodical rest from labor is necessary to the well-being of the body; and those who are not convinced of it by these proofs, would not be by anything additional. Nature is not more explicit in demanding rest than is Revelation. *Remember the Sabbath Day to keep it holy.* There is no use in attempting to explain this away. The Almighty interpreted it himself, when *He rested on the seventh day from all His work.* This article is for those who believe in the Bible. To such, one plain explicit text is as good as a thousand. The man who wants various texts of Scripture to convince him of the truth of a doctrine or practice, would not *be persuaded though one rose from the dead.* We, therefore, address ourselves to intelligent, Bible-reading people; and we trust that such believe firmly that the Sabbath ought to be observed as a day of rest. As it is therefore necessary to the health and preservation of the body to give it one day's rest in seven, it comes within the sphere of a *Journal of Health* to make it practical, by stating some of the ways by which the more proper and general observance of the Sabbath may be brought about, especially as to large cities. Let us then begin at the beginning, and address ourselves to single-hearted, self-denying Christian people—that happiest class of all, which needs no other warrant or authority for any line of practice than this: *The Bible says it.* We trust we may take it for granted, that there are from two to a hundred such in every evangelical church in the land; and if these were to begin aright, with themselves, and with their children, and then with their servants, and those employed by or dependent on them, it need not be long before an impression would be made for good, which would soon double upon itself, and pervade our common country.

In our plan of beginning, we do not calculate upon any large number agreeing with us; but a beginning must be made; and to make it clear, let it be radical—let the line of departure be broad and distinct. We start with that little principle involved in the case of PAUL, when he said he would not eat meat, if it offended; that is to say, he was willing to sacrifice his rights for purposes of conciliation, or if the cause of religion was promoted thereby: in other words, we will begin with the spirit of a generous, whole-souled self-denial.

1. Let nothing in print be read in your household on Sunday, except the Bible, the Prayer-Book, the Hymn-Book, the Catechism, and Scripture Concordance—all others are the words of fallible men, and have in them, more or less, a taint of error or sin.

2. Have no cooking done in your house on Rest-Day, except the making of coffee and tea. This is allowed; because, as a physician, we know that persons accustomed to these will be physically incapacitated for bodily or mental enjoyment, if taking them is omitted. We grew up with such an observance, and know it is practicable.

3. Walk to church, if it is within a mile of your dwelling, unless age and infirmity prevent; and in that case, we are almost inclined to think it would be better not to go to church at all, so as not to offend the weak or captious; it can be made up by the minister having a prayer-meeting or lecture at such household. Our minister used to do this (JOHN McFARLAND, the pupil of the elder Mason); that is, he would hold such meetings at the houses of those who could not attend church.

4. Allow no milk, ice, butcher, or other provision cart to stop at your door on Sundays. This is practicable in all seasons—*easily* practicable, as we personally know. The greatest difficulty is about the milk, but if BORDEN'S *Condensed Milk* is used,—the purest and best in the world, next to milking your own cows—milk need be taken but twice a week in Summer, and once in Winter.

6. Visit nobody, except in sickness, and allow nobody to visit you.

6. Attend but two religious services on the Sabbath day; more is a waste, both to minister and people.

7. Take no Sunday walks or drives, on the pretence of its being good for the health. People who walk or recreate for their health on Sundays, do not do it on any other day of the week.

8. In process of time, allow no train of cars to arrive or depart within the city limits, nor any vessel to debark her freight, or to load up on Sundays. Ten years ago, there was such an ordinance in New Orleans—a city supposed by many Northerners to be not much better than Sodom (they would know better if they lived there, *and would act, while there, with as much decency as they were accustomed to do at home*). The plan worked well, and there was no difficulty in procuring its observance, as far as we know. The ordinance went into operation from ten o'clock on Sunday mornings.

9. Procure the closing of every business place, where money is given or received—apothecary shops and hotels excepted. There would be no difficulty worth naming in securing this last item, if the religious and respectable class of citizens would unite upon it.

The nine particulars named would be a starting-point, and if rigidly and continuously adhered to, would soon make a Sabbath in New York what it used to be, a short half century ago. That it would require self-denial, trouble, and heroic persistence, we do not deny; but, considering the hold which

Evangelical Christianity has on public sentiment, we believe it is practicable to have a more consistent observance of the Sabbath day than that which now prevails. Who will be the pioneer in this thing, as a minister, elder, deacon, church member? However obscure he may be, we believe a consistent and persistent devotion to the line of right in this thing, would kindle like a spark in the dryest wood, and conflagrate the world. Why not? Leaven is diffusive—the Bible our authority, and Omnipotence our Leader.

These suggestions are made with reference to a "Report" handed us, *For promoting the better observance of the Sabbath.* We have not entered into an extended argument as to the points proposed. We have rather taken the things for granted, as we are addressing Bible readers. But, in a medical point of view, we express it as our conviction, that a plainer kind of eating on Sundays would give rest to the stomach, by taking away the temptation to eat largely. But if we have better eating on Sundays than on other days, we will eat more; while by exercising less, by a great deal, than on week-days, we do a double wrong to the system, and thus make ourselves more like gorged anacondas than anything else; and in proportion unfitted for religious duty, or for anything else that is elevating and refining—offering a somnolent worship, instead of that which is spiritual and life-giving.

There need be no tedious sameness in a Sabbath when a family is confined to such books as we have named, and to only two public services. The singing of psalms, and hymns, and spiritual songs, might be made an interlude or recreation to the more serious reading or conversation. There is nothing more delightful in the present, and in the reminiscence, than the spectacle of a Christian family sitting around the fire in Winter time, singing the various parts of some familiar hymn. Any music is elevating, and especially sacred music; and it is a sign of refinement in any family to see a "note-book" lying around, or a newspaper devoted to the harmony of sweet sounds,

In addition to all this, there is an inexhaustible source of instruction for children in the histories of Old Testament times, which, if studied and enlarged upon by an intelligent parent, would rivet the attention of our children from one year's end to another, and so fix upon their memories Bible histories as to make them classical, and withal instructive for life. What more so than Jacob's leaving home, to carve out his own fortunes! Joseph's temptation and fidelity; the history of Moses; the Prophet and the bears in the wood; of Hannah and Samuel; of the son of Jesse; the attachment of David and Jonathan; of the mean-hearted slanderer and curser Shimei; and a multitude of others, all fraught with lessons of constant application in practical life. By thus interspersing the hours of the Sabbath with the repetition of instructive histories, founded in the Bible, by family singing, and cheerful, affectionate conversation, whetting up one another's memories and attentions by questions about the sermon heard, Sunday may be a day looked forward to, as really a day of release and rest.

We cannot see why the Sabbath may not be kept in cities as well as in country villages; in many of which *all* worldly occupations are suspended without the aid of any law, other than an intelligent public sentiment. One of the greatest obstacles we see in bringing about the changes desired, will be found in unprincipled editors and flash preachers—men whose ambition is to court the titter of the mob, and to be considered *liberal* in their views; who are always prating about "Puritanical Practices," "Old Fogyism," and other clap-trap phrases.

Money is Power. The men who are either members of churches, or are habitual attendants on public worship, are very generally men of thrift. We believe three-fourths of the wealth of our large cities is in the hands of such men and their families These are the persons who own half the real estate of our city, hold half the stock and bonds of our great corporations, banks, railroads, and shipping; and, consequently, have it in their power, to a great extent, if so disposed, to put an end to shipping and railroad Sunday desecration; to put an end to the occupancy of their property as drinking establishments, gambling saloons, and for purposes still more infamous. If all leases for grocery stores, eating houses, dram shops, and the like, had a clause of forfeiture, if ever found open on Sundays, a great advance would be made in a good direction. For the twenty-four hours ending on Sunday night, a larger amount of crime and beastliness is committed than in any other twenty-four hours of the week. If every liquor-selling place could be closed at ten o'clock on Saturday night, and kept closed until Monday morning, an amount of human degradation and deviltry would be prevented beyond conception.

One of the largest and costliest buildings now in progress in New York is from the proceeds, in very great part, of the rent of first-class houses of prostitution. Several of the steamboats which take out Sunday excursionists are owned by men who are reputed respectable. Let a wholesome public sentiment frown on these things, and they would in time be abated. But to manufacture that sentiment, we know of no more efficient plan than the one we have proposed: let each church member officer, and minister, begin at home, and abolish all unholy Sunday reading, abolish Sunday cookery, Sunday visiting, and Sunday recreation, and in every way possible countenance and encourage by your patronage the men and institutions which most respect the Sabbath; and, in due time, rail travel would cease; the Sunday press would die of inanition; the dram-shop would be closed, and the insufferable yellings of milkmen, with the racket of provision-carts, would be put an end to; and in the language of good old PETER STUYVESANT (whose pear-tree, planted over two hundred years ago, still blooms, and blossoms, and bears its fruit, within a few blocks of our dwelling), "we would prevent the curse of God (instead of his blessing) falling on us and our good inhabitants;" and by making Sunday truly a day of rest, both body and mind would be recuperated, we would enter more vigorously upon the duties of the week following, would perform them with greater alacrity, and with greater fidelity would execute them, thus promoting length of days as well as happifying them.

